# Influence of Bi^3+^ Doping on Electrochemical Properties of Ti/Sb-SnO_2_/PbO_2_ Electrode for Zinc Electrowinning

**DOI:** 10.3390/molecules29174062

**Published:** 2024-08-27

**Authors:** Jia Wu, Xuanqi Kang, Shuangwen Xu, Zhen Wei, Shangyuan Xu, Kang Liu, Qing Feng, Bo Jia, Yunhai Wang

**Affiliations:** 1 Xi’an Taijin New Energy & Materials Sci-Tech Co., Ltd., Xi’an 710016, China; kangsiqiqi@163.com (X.K.); xu_shuangwen@163.com (S.X.); wzgryx1991@163.com (Z.W.); xushangyuanx@163.com (S.X.); liukang5410@163.com (K.L.); fengqing0715@126.com (Q.F.);; 2 State Key Laboratory of Multiphase Flow in Power Engineering, Department of Environmental Science and Engineering, Xi’an Jiao tong University, Xi’an 710049, China; wang.yuanhai@mail.xjtu.edu.cn

**Keywords:** Ti/Sb-SnO_2_/PbO_2_ electrode, Bi^3+^ doping, electrocatalytic activity, zinc electrowinning

## Abstract

Bi^3+^ doped Ti/Sb-SnO_2_/PbO_2_ electrode materials were fabricated by electrodeposition to improve their electrochemical performance in zinc electrowinning. The surface morphology, chemical composition, and hydrophilicity of the as-prepared electrodes were characterized using scanning electron microscopy (SEM), energy dispersive X-ray spectroscopy (EDS), X-ray diffraction (XRD), X-ray photoelectron spectroscopy (XPS), and contact angle. An electrochemical measurement and an accelerated lifetime experiment were also conducted to investigate the electrocatalytic performance and stability of the electrodes. The results show that the Bi^3+^ modification electrode has an important effect on the coating morphology, the crystal structure, the surface hydrophilicity, the electrocatalytic activity, and the stability. The electrode prepared from the solution containing 2 mmol·L^−1^ Bi(NO_3_)_3_ (marked as the Ti/Sb-SnO_2_/2Bi-PbO_2_ electrode) exhibits the best hydrophilicity performance (θ = 21.6°) and the longest service life (1196 h). During the electrochemical characterization analysis, the Ti/Sb-SnO_2_/2Bi-PbO_2_ electrode showed the highest oxygen evolution activity, which can be attributed to it having the highest electroactive surface (q_T_* = 21.20 C·cm^−2^) and the best charge-transfer efficiency. The DFT calculation demonstrated that the doping of Bi^3+^ leads to a decrease in the OER reaction barrier and an increase in the DOS of the electrode, which further enhances the catalytic activity and the conductivity of the electrode. Moreover, the simulated zinc electrowinning experiment demonstrated that the Ti/Sb-SnO_2_/2Bi-PbO_2_ electrode consumes less energy than other electrodes. Therefore, it is expected that the Bi^3+^ modified electrode will become a very promising electrode material for zinc electrowinning in the future.

## 1. Introduction

Zinc is widely used in mechanical processing, chemical industry, medicine, aerospace, electronics, and other fields [1,2,3]. Currently, approximately 85% of the zinc used in different industries is obtained by hydrometallurgy [4,5,6]. Compared to pyrometallurgy, hydrometallurgy has the advantages of a large production capacity, high efficiency, and low environmental pollution. Zinc electrowinning is considered an important part of the hydrometallurgy process; it occupies more than 80% of the total energy of zinc hydrometallurgy [7,8,9]. The electrode material employed in zinc electrowinning has a significant impact on various parameters, including current efficiency, energy consumption, production cost, quality of the zinc, etc. [10,11,12]. At present, lead-based electrodes have been widely used for zinc electrowinning [13,14,15]. However, there are some problems which arise when using this anode, including poor corrosion resistance, easy distortion, high energy consumption, and the cathode zinc being susceptible to Pb contamination [16,17,18]. Therefore, novel anode materials (Ti-based metal oxide, Al/PbO_2_, SS/PbO_2_) with suitable stability, high electrical conductivity, and good electrocatalytic activity have attracted much attention [19,20,21]. Among the anode materials mentioned above, Ti-PbO_2_ anodes exhibit superior properties, including strong size stability, long service life, excellent corrosion resistance, low production cost, and high electrocatalytic activity [22,23,24]. However, the Ti-PbO_2_ electrode is not ideal for actual application. The main drawbacks of the Ti-PbO_2_ electrode for zinc electrowinning are the high oxygen evolution reaction (OER), the large internal stress, and the short service life. To solve these problems, the doping of various elements and composites in electrolytes including Ce^4+^, Ag^+^, CeO_2_, Co_3_O_4_, and RuO_2_ has been employed in the PbO_2_ coating, which has also been demonstrated to be an effective approach [25,26,27,28,29,30]. With the rapid progress of technology, green and sustainable development has become the target of zinc hydrometallurgy. Thus, researchers have given much interest to the anode materials with long lifetimes, high activity, and low energy consumption.

**Figure 1:**
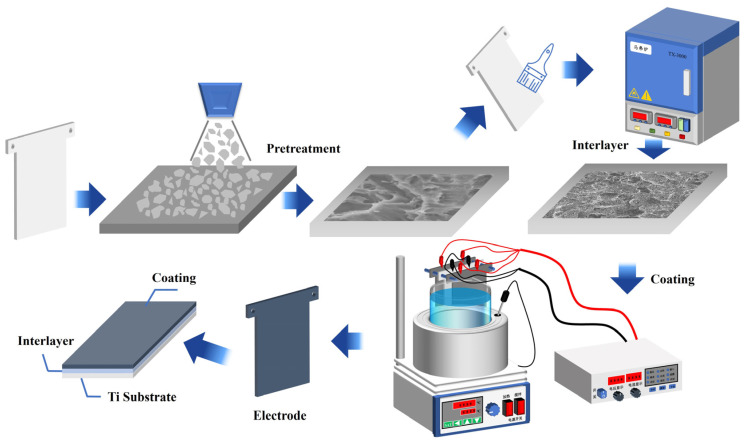
A schematic diagram of electrode preparation.

The non-toxic Bi^3+^ (103 pm) and Pb^2+^ (119 pm) are known to have very similar ionic radii. Therefore, their oxides easily form solid solutions that improve electrode surface adhesion and increase the electrode conductivity [31,32,33]. It is worth noting that the presence of Bi^3+^ ions influences the kinetics of PbO_2_ electrodeposition, resulting in the morphological modification of the PbO_2_ coating. Moreover, the phase composition is also influenced by the incorporation of Bi^3+^ ions [34,35]. Therefore, in this work, Bi^3+^ ions were introduced into the Ti/Sb-SnO_2_/PbO_2_ electrode by electrodeposition to improve the anode stability and decrease the OER of the electrode. The principal diagram of the Bi^3+^ modification electrode is shown in Figure 1. Meanwhile, the effect of Bi^3+^ ions with various concentrations on the crystal structure and surface morphology was also studied in detail. The electrochemical properties of the as-fabricated anodes were investigated using cyclic voltammetry (CV), linear sweep voltammetry (LSV), and electrochemical impedance spectroscopy (EIS).

## 2. Results and Discussion

### 2.1. Surface Morphology and Crystal Structure

**Figure 2:**
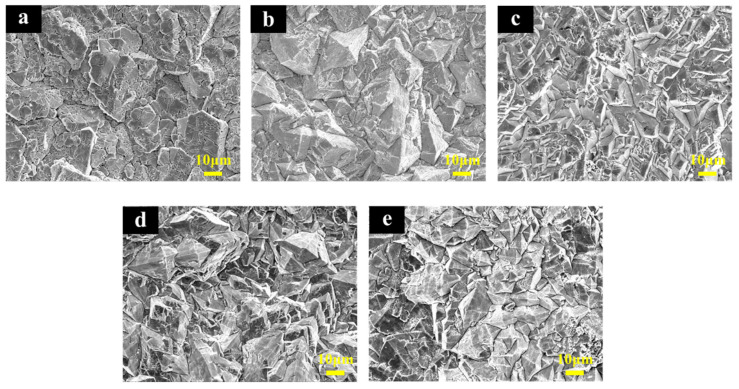
SEM of the electrodes: (**a**) Ti/Sb-SnO_2_/PbO_2_; (**b**) Ti/Sb-SnO_2_/1Bi-PbO_2_; (**c**) Ti/Sb-SnO_2_/2Bi-PbO_2_; (**d**) Ti/Sb-SnO_2_/3Bi-PbO_2_; and (**e**) Ti/Sb-SnO_2_/5Bi-PbO_2_.

**Figure 3:**
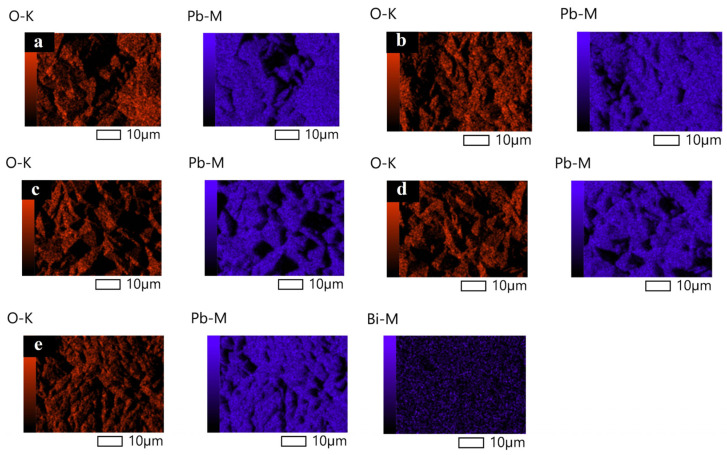
EDS Mapping of the electrodes: (**a**) Ti/Sb-SnO_2_/PbO_2_; (**b**) Ti/Sb-SnO_2_/1Bi-PbO_2_; (**c**) Ti/Sb-SnO_2_/2Bi-PbO_2_; (**d**) Ti/Sb-SnO_2_/3Bi-PbO_2_; and (**e**) Ti/Sb-SnO_2_/5Bi-PbO_2_.

The surface morphologies of Ti/Sb-SnO_2_/Bi-PbO_2_ electrodes prepared at different Bi^3+^ concentrations are shown in Figure 2. It can be seen that the addition of Bi^3+^ into a PbO_2_ coating significantly affects the coating morphology. The film becomes denser and the particles become smaller with the introduction of Bi^3+^. We suggest that the doping of Bi^3+^ into the PbO_2_ coating could improve the coating structure effectively. This can be attributed to the formation of heterogeneous nuclei of bismuth oxide, which increases the number of crystal nuclei and hinders the growth of particles during the crystallization of the coating [36]. The particles on the electrode surface exhibit the smallest size when the Bi(NO_3_)_3_ concentration is 2 mmol·L^−1^. However, a further increase in Bi(NO_3_)_3_ concentration is not beneficial for the decrease in the size of the particles. The corresponding EDS mapping images of the Bi, O, and Pb elements are shown in Figure 3. We found that the Bi element is not detected in Ti/Sb-SnO_2_/PbO_2_, Ti/Sb-SnO_2_/1Bi-PbO_2_, Ti/Sb-SnO_2_/2Bi-PbO_2_, or Ti/Sb-SnO_2_/3Bi-PbO_2,_ due to the detection limits of the instrument. However, the Bi element is evenly distributed on the Ti/Sb-SnO_2_/5Bi-PbO_2_ electrode surface.

**Figure 4:**
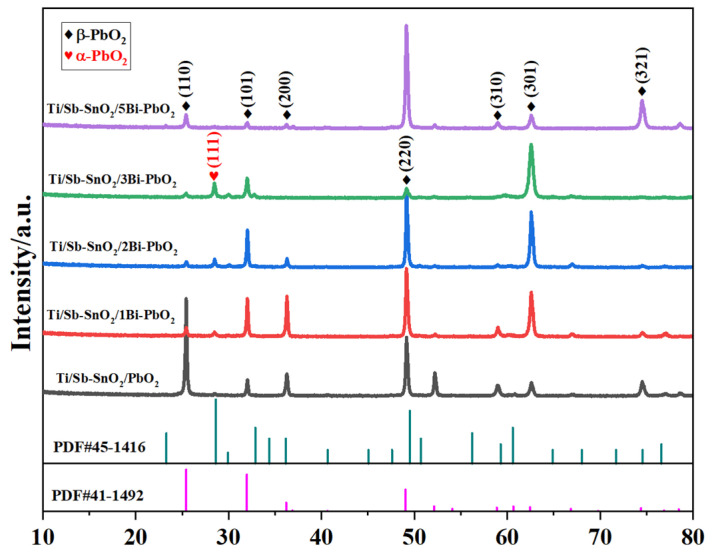
XRD of the as−prepared electrodes with different Bi(NO_3_)_3_ concentrations.

Figure 4 shows the XRD patterns of the as-prepared electrodes with a variation Bi^3+^concentration. As depicted in Figure 4, the diffraction peaks observed at 2θ = 25.4°, 32.0°, 36.2°, 52.1°, 58.9°, 62.5°, 74.4° were assigned to the (110), (101), (200), (220), (310), (301), and (321) planes of β-PbO_2_ (JCPDS#41-1492). The diffraction peaks observed at 2θ = 28.6° were assigned to the (111) plane of α-PbO_2_ (JCPDS#45-1416). It was found that all the electrodes exhibited reflections of β-PbO_2_ and α-PbO_2_. No peaks corresponding to bismuth oxide were detected in the XRD pattern of Ti/Sb-SnO_2_/xBi-PbO_2_, indicating that the doping amount of bismuth is small and lower than the detective limit of XRD. It should be noted that the crystalline of α-PbO_2_ and the main plane of β-PbO_2_ varied with the concentration of Bi^3+^ doping. The main planes of Ti/Sb-SnO_2_/PbO_2_ were (110) and (220). After doping with a low-concentration Bi(NO_3_)_3_ (≤2 mmol·L^−1^), the diffraction peaks of crystal planes (101) and (301) were enhanced, indicating that the directions of crystal planes (101) and (301) were preferentially selected. However, Bi(NO_3_)_3_ content which was too high was not favorable for the growth of crystal planes (101) and (301). When the concentration of Bi(NO_3_)_3_ was 5 mmol·L^−1^, the main plane of Ti/Sb-SnO_2_/5Bi-PbO_2_ was (220). These results imply that the doping concentration of Bi(NO_3_)_3_ affects the selective growth of β-PbO_2_ crystal planes.

### 2.2. XPS Analysis

**Figure 5:**
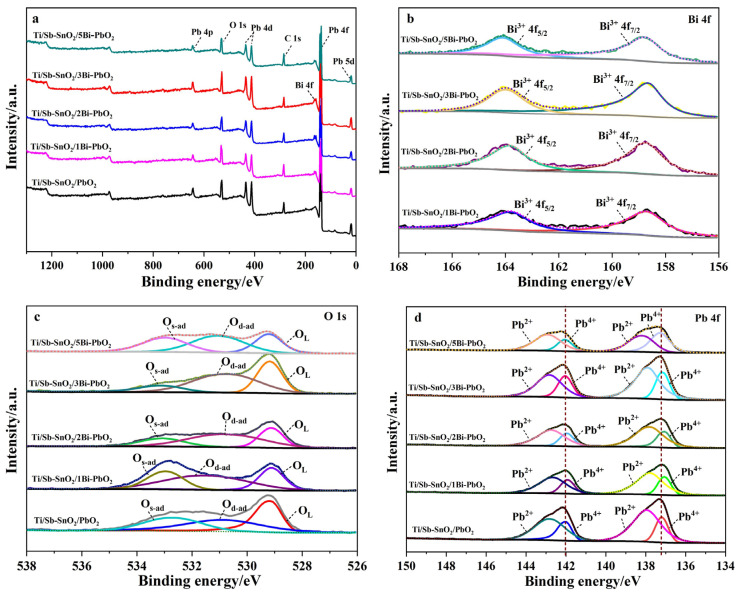
XPS spectra of the as−prepared electrode: (**a**) survey; (**b**) Pb 4f; (**c**) O 1s; and (**d**) Bi 4f.

An XPS measurement is performed to further analyze the chemical states of the elements supported on the as-synthesized electrodes. Figure 5a reveals the survey spectrum of the as-prepared electrodes, which exhibit Pb, O, and C elemental signals. Moreover, Bi^3+^ elemental peaks can be observed for Ti/Sb-SnO_2_/xBi-PbO_2_ (x = 1, 2, 3, 5), suggesting that Bi ions are successfully doped in the PbO_2_ coating. Figure 5b displays the 4f core-level spectra recorded for Bi. The peaks at 158.7 and 163.8 eV correspond to Bi 4f_7/2_ and Bi 4f_5/2_, respectively, which confirms the presence of the Bi^3+^ species. Figure 5c shows the O 1s core level of the as-prepared electrodes, which can be deconvoluted into three characteristic peaks: lattice oxygen species (~529.2 eV for O_L_), surface oxygen vacancies and adsorbed oxygen (530.5–531.7 eV for O_d-ad_), and surface adsorbed molecular water (~532.9 eV for O_s-ad_), as listed in Table 1. It should be noted that the formation of surface oxygen vacancies is closely related to the highly oxidative oxygen species and is active for catalyzing OER [37]. From Table 1, it can be found that the proportions of O_d-ad_ present on the electrode surface first increase with the Bi^3+^ doping content and then decrease with a further increase of the Bi^3+^ doping content. The maximum proportion of O_d-ad_ is obtained when the concentration of Bi(NO_3_)_3_ is 2 mmol·L^−1^, indicating the best OER activity.

To elucidate the impact of doping on PbO_2_ electrodeposition, the Pb 4f spectrum was performed, as shown in Figure 5d. In the Ti/Sb-SnO_2_/PbO_2_ electrode, peaks of 142.01 eV and 137.20 eV correspond to the Pb^4+^, while peaks of 142.82 eV and 137.96 eV correspond to Pb^2+^, as shown in Table 2. The simultaneous presence of Pb^4+^ and Pb^2+^ in the electrode indicates the formation of non-stoichiometric PbO_2_ during the electrodeposition. After doping with Bi^3+^, the binding-energy peak of Pb^4+^ shifted, suggesting a strong interaction between Bi^3+^ and Pb [38]. This converts some PbO_2_ into a lower-valence compound, promoting the proportion of non-stoichiometric PbO_2_, thus improving the electrode conductivity.

### 2.3. Hydrophilicity Analysis

**Figure 6:**
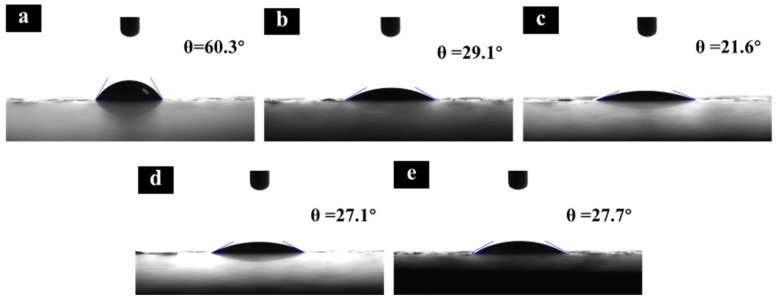
Contact angles (θ) of the as−prepared electrode: (**a**) Ti/Sb-SnO_2_/PbO_2_; (**b**) Ti/Sb-SnO_2_/1Bi-PbO_2_; (**c**) Ti/Sb-SnO_2_/2Bi-PbO_2_; (**d**) Ti/Sb-SnO_2_/3Bi-PbO_2_; and (**e**) Ti/Sb-SnO_2_/5Bi-PbO_2_.

The hydrophilicity of the surfaces has been shown to promote the charge-transfer rate between the electrolytes and the electrodes, enhancing the oxygen evolution reaction activity. Therefore, the contact angle of the as-synthesized electrodes was measured to evaluate the surface wettability. As shown in Figure 6, the contact angles (θ) in Ti/Sb-SnO_2_/PbO_2_, Ti/Sb-SnO_2_/1Bi-PbO_2_, Ti/Sb-SnO_2_/2Bi-PbO_2_, Ti/Sb-SnO_2_/3Bi-PbO_2_, and Ti/Sb-SnO_2_/5Bi-PbO_2_ were 60.3°, 29.1°, 21.6°, 27.1°, and 27.7°, respectively. It can be found that much smaller θ are observed for the Ti/Sb-SnO_2_/xBi-PbO_2_ electrode compared to pure Ti/Sb-SnO_2_/PbO_2_ (60.3°), indicating the enhanced compatibility and affinity of the electrode for Bi^3+^ doping, which in turn leads to enhancement of their catalytic activity in the OER. When the concentration of Bi(NO_3_)_3_ further increases, the contact angles of Ti/Sb-SnO_2_/3Bi-PbO_2_ and Ti/Sb-SnO_2_/5Bi-PbO_2_ are slightly higher than that of Ti/Sb-SnO_2_/2Bi-PbO_2_, which may be ascribed to the change of crystal planes for the electrode.

### 2.4. Electrochemical Property

**Figure 7:**
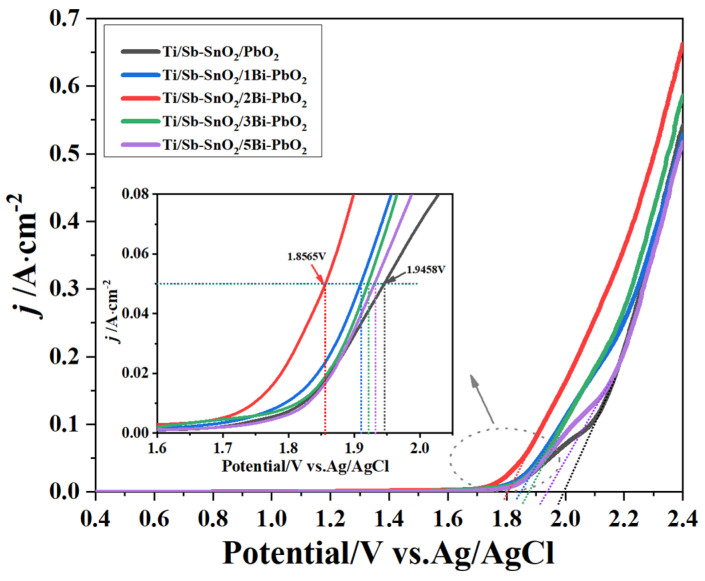
LSV curves of the as−prepared electrode.

An LSV test was performed to obtain the oxygen evolution potentials (OEP) of the as-prepared electrodes, as displayed in Figure 7. The results show that the OEP of the electrode decreases with the addition of Bi(NO_3_)_3_. The onset potentials for OEP on Ti/Sb-SnO_2_/PbO_2_, Ti/Sb-SnO_2_/1Bi-PbO_2_, Ti/Sb-SnO_2_/2Bi-PbO_2_, Ti/Sb-SnO_2_/3Bi-PbO_2_, and Ti/Sb-SnO_2_/5Bi-PbO_2_ are 1.99 V, 1.84 V, 1.80 V, 1.87 V, and 1.93 V (vs. Ag/AgCl), respectively. This implies that the introduction of Bi^3+^ provides more active sites for the oxygen evolution reaction, and that the electrocatalytic activity gradually increased. Furthermore, as shown in Figure 7, the potential of the Ti/Sb-SnO_2_/2Bi-PbO_2_ electrode is found to be 89 mV lower than that of the Ti/Sb-SnO_2_/PbO_2_ electrode (500 A/m^2^), indicating that the addition of Bi^3+^ can effectively improve the electrocatalytic activity of the PbO_2_ electrode. However, when the concentration was greater than 2 mmol·L^−1^, there was little difference in potential, and the increase of Bi^3+^ resulted in the excessive increase of PbO_2_ crystal grain. This may cause a decrease in surface roughness and a decrease in the active regions involved in the reaction.

**Figure 8:**
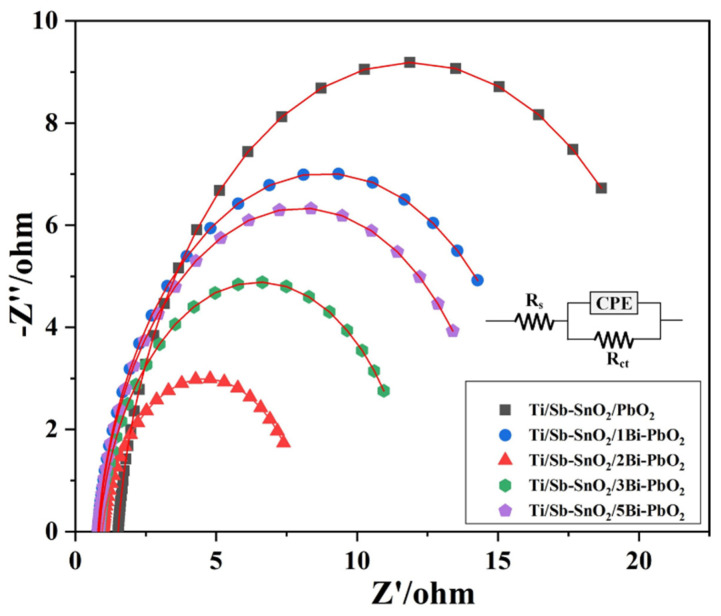
EIS curves of the as-prepared electrode.

EIS measurements were conducted to further explain the influence of Bi(NO_3_)_3_ on the oxygen evolution capacity of the PbO_2_ coatings. Figure 8 exhibits the electrochemical impedance of PbO_2_ electrodes with an applied potential of 1.90 V vs. Ag/AgCl, which corresponds to the oxygen evolution zone. In the equivalent circuits, R_s_, R_ct_, and constant phase angle element (CPE) represent the ohmic resistance, the charge-transfer resistance, and the double-layer capacitance. The R_s_ include the electrolyte resistance and the active material resistance. R_ct_ reflects the oxygen release reaction activity. The CPE was employed to substitute the capacitance when n is between 0.9 and 1. The simulation data for each parameter are shown in Table 3. The R_ct_ value of the Ti/Sb-SnO_2_/2Bi-PbO_2_ electrode is 7.30 ohm, which is lower than those of the other electrodes. The Ti/Sb-SnO_2_/PbO_2_ electrode exhibits the largest R_ct_ value of 20.80 ohm. Furthermore, the R_ct_ value gradually reduced with the addition of Bi(NO_3_)_3_. The minimum value was obtained when the Bi(NO_3_)_3_ concentration was 2 mmol·L^−1^. On the contrary, the R_ct_ value increased slightly when the Bi(NO_3_)_3_ concentration was further increased, indicating that the modification of Bi^3+^ could significantly increase the catalytic activity of the coating, and that the optimum Bi(NO_3_)_3_ concentration is 2 mmol·L^−1^. It can be inferred that high oxygen evolution activity and low R_ct_ are in favor of the catalytic activity. Earlier research has observed that oxygen evolution activity depends on the active site of the PbO_2_ coating, and that large active sites imply good reactivity [39].

**Figure 9:**
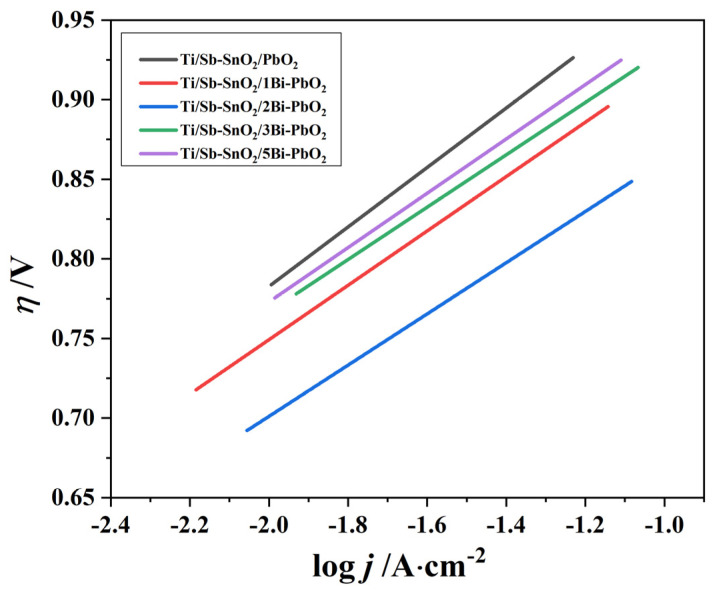
Tafel curves of the as−prepared electrode.

The oxygen evolution activity can be determined by comparing the oxygen evolution overpotentials. Figure 9 displays the Tafel curve of the as-prepared electrodes. The oxygen evolution overpotential (η) was employed to investigate the oxygen evolution overpotential (η) of the electrode material and it was obtained according to Formula (2) [40]:

η = E + 0.224 − 1.241 − iR_s_ (1)

where E represents the potential for the reference electrode (Ag/AgCl), measured by the anodic polarization curve; 1.241 V is the standard potential in the measurement system (50 g·L^−1^ Zn^2+^ + 150 g·L^−1^ H_2_SO_4_); 0.224V is the potential of the Ag/AgCl relative to the standard hydrogen electrode; and R_s_ is the electrolyte resistance between the reference electrode and the working electrode.

The η and the current density j exhibit a semi-logarithmic relationship, as shown in Formulas (3)–(5):

η = a + blgj; (2)

a = −2.3(RT/βnF)lgJ_0_; (3)

b = 2.3RT/βnF, (4)

where a and b are Tafel parameters during anodic polarization and j is the measured current density at the relative potential.

The corresponding overpotentials and kinetic parameters for the as-synthesized electrodes are given in Table 4. We found that under the conditions of 500 and 1000 A·m^−2^, the Ti/Sb-SnO_2_/2Bi-PbO_2_ electrode exhibited the lowest η values (0.814 V and 0.862 V), indicating the high probability of oxygen evolution reaction. When the concentration of Bi(NO_3_)_3_ increased from 0 to 2 mmol·L^−1^, the η gradually decreased, and the η began to increase at 3 mmol·L^−1^. The electrocatalytic performance of the electrode is closely related to the Tafel parameters. Usually, parameter a reflects the cell voltage and the cell voltage decreases with the decreasing value, which means a small energy consumption. Table 4 shows that the values of the electrodes Ti/Sb-SnO_2_/PbO_2_ and Ti/Sb-SnO_2_/2Bi-PbO_2_ are 1.141 V and 1.023 V, respectively. The variation of a value and η follows the same tendency. Moreover, the b value represents the overpotential of the material. The smaller b value denotes high catalytic activity. The electrode fabricated at 2 mmol·L^−1^ Bi(NO_3_)_3_ exhibited the lowest b value, indicating the highest catalytic activity for an oxygen evolution reaction. This consequence is in accord with the EIS results.

**Figure 10:**
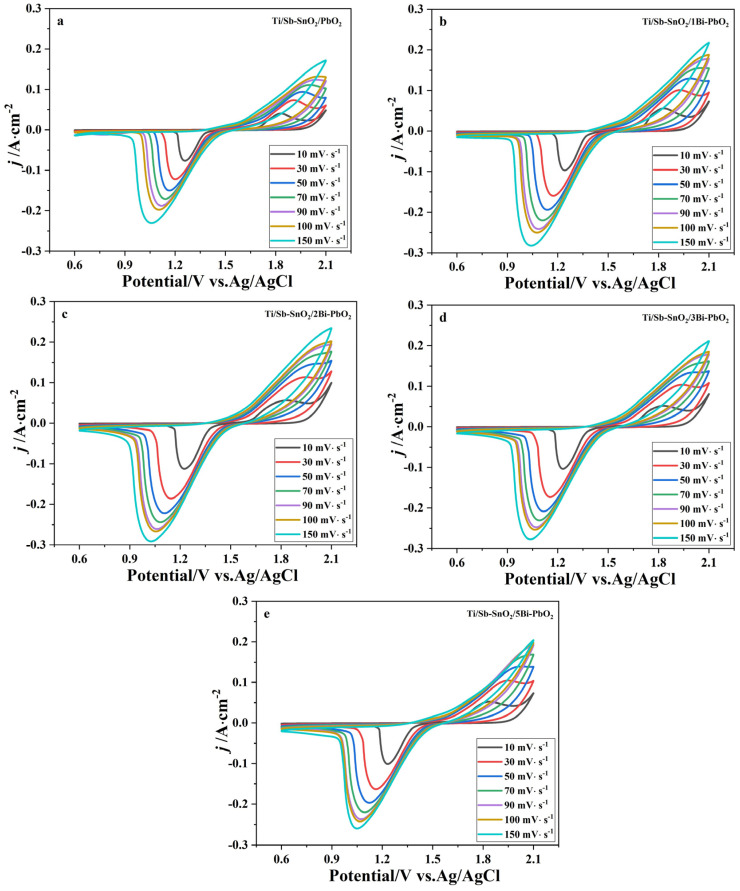
CV curves of the as-prepared electrode under different scan rates: (**a**) Ti/Sb-SnO_2_/PbO_2_; (**b**) Ti/Sb-SnO_2_/1Bi-PbO_2_; (**c**) Ti/Sb-SnO_2_/2Bi-PbO_2_; (**d**) Ti/Sb-SnO_2_/3Bi-PbO_2_; and (**e**) Ti/Sb-SnO_2_/5Bi-PbO_2_.

**Figure 11:**
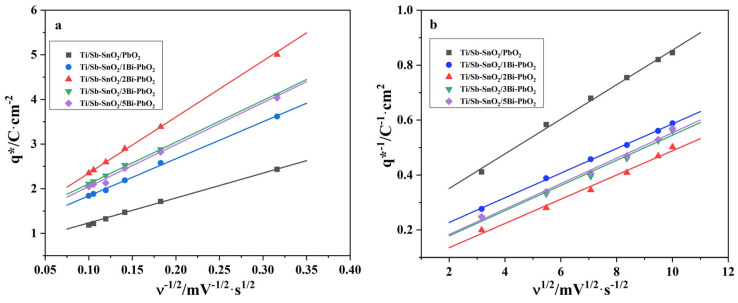
(**a**) Extrapolation of q_o_* for the as−prepared electrode from the representation of q* versus ν^−1/2^. (**b**) Extrapolation of q_T_* for the as−prepared electrode from the representation of (q*)^−1^ versus ν^1/2^.

The amount of voltammetric charge (q*) is related to the actual surface area and number of active sites, which can reflect the electrochemical activity of the electrode [41]. It is found that the q* depends on the potential scan rate (ν). Therefore, q* can be obtained by integrating the CV curve. Figure 10 displays the CV curve of the as-prepared electrodes under various potential scan rates. According to the literature, total voltammetric charge (q_T_*), outer voltammetric charge (q_o_*), and inner voltammetric charge (q_i_*) are calculated by the Equations (6)–(8), respectively.

q* = q_o_* + k_1_ν^−1/2^; (5)

q*^−1^ = q_T_*^−1^ + k_2_ν^1/2^; (6)

q_T_* = q_o_* + q_i_*, (7)

where k_1_ and k_2_ are constants; ν is the scan rate. It is reported that q_T_* and q_i_* are related to the entire electroactive surface and to less accessible electroactive sites, respectively. The electrochemical porosity can be obtained by computing q_i_*/q_T_*. Figure 11a shows that q* is linearly related to ν^−1/2^, and q_o_* can be obtained from the extrapolation to ν^−1/2^ = 0. Moreover, q*^−1^ is also linearly related to ν^1/2^, and extrapolation of these straight lines to ν^1/2^ = 0 gives q_T_*, as shown in Figure 11b.

Table 5 summarizes the total, outer, and inner charges obtained for the as-prepared electrodes. The q_T_* of Ti/Sb-SnO_2_/PbO_2_, Ti/Sb-SnO_2_/1Bi-PbO_2_, Ti/Sb-SnO_2_/2Bi-PbO_2_, Ti/Sb-SnO_2_/3Bi-PbO_2_, and Ti/Sb-SnO_2_/5Bi-PbO_2_ is 4.44 C·cm^−2^, 7.25 C·cm^−2^, 21.20 C·cm^−2^, 11.49 C·cm^−2^, and 11.01 C·cm^−2^, respectively. It can be seen that the doping of Bi^3+^ could greatly improve the electroactive surface of the electrodes. The electrode prepared at 2 mmol·L^−1^ Bi(NO_3_)_3_ exhibited the highest q_T_*, suggesting a higher electroactive surface. However, excessive doping resulted in a decrease in q_T_*, which is not beneficial for the increase in the electroactive surface. Moreover, the ratio of q_i_*/q_T_* for Ti/Sb-SnO_2_/2Bi-PbO_2_ electrodes is higher than the other electrodes, which is in favor of an increase in the active site. This result is consistent with the Tafel tendency.

### 2.5. DFT Calculations

**Figure 12:**
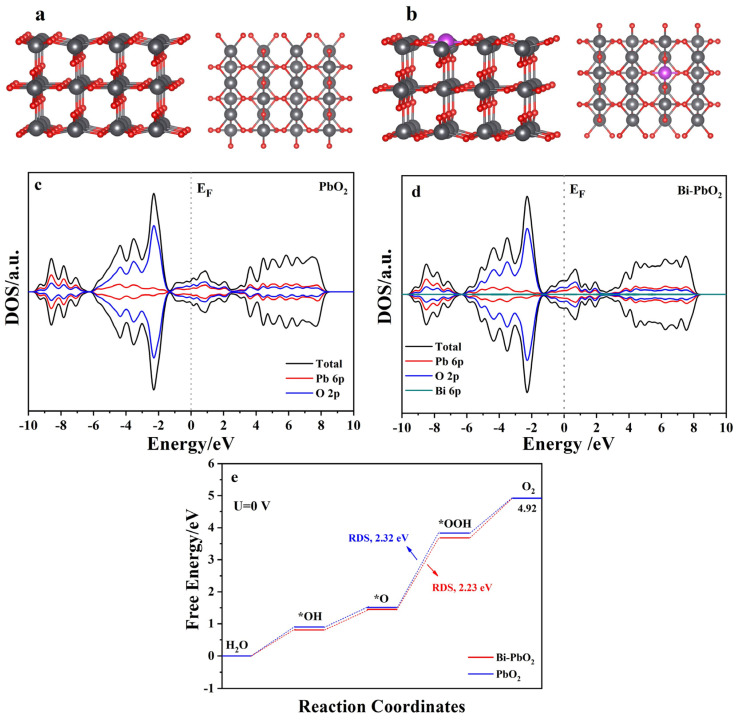
(**a**,**b**) Structural diagrams of PbO_2_ and Bi−PbO_2_, respectively (black, red, and purple spheres represent Pb, O, and Bi atoms, respectively); (**c**,**d**) Density of States for PbO_2_ and Bi−PbO_2_, respectively; (**e**) free−energy diagrams for OER in PbO_2_ and Bi−PbO_2_, respectively (* represent the ctive site on the surface model).

To verify the effect of Bi^3+^ doping on PbO_2_, the Density of State (DOS) and the OER activity of the Bi atom were studied using DFT calculations (Figure 12). Figure 12a,b displays the Schematic diagram of the structures. Figure 12c,d shows the DOS of the PbO_2_ and Bi-PbO_2_ electrodes. We observe that Bi doping could slightly improve the value of the DOS at the Fermi level. Thus, the introduction of Bi can improve electrode conductivity. The Gibbs free energies of the PbO_2_ and Bi-PbO_2_ at each step of their OER reactions are also calculated (Figure 12e). According to the literature [42,43,44], OER is a four-electron step process. Oxygen intermediates include *OH, *O, and *OOH. The largest Gibbs free energy is usually considered as the rate-determining step (RDS), which determines the electrode OER electrocatalytic activity. As shown in Figure 12e, the conversion of *O to *OOH is the RDS of the PbO_2_ and Bi-PbO_2_. In the RDS procedure, the energy barrier of PbO_2_ and Bi-PbO_2_ is 2.32 eV and 2.23 eV, respectively. Therefore, the electrocatalytic activity of the Bi-PbO_2_ electrodes is higher than that of the PbO_2_ electrode. The difference in energy barrier can be ascribed to the introduction of the Bi atom and the enhanced catalytic sites. Combined with theoretical calculations, the Bi doping leads to a decrease in the OER reaction barrier and an increase in the specific surface area of the electrode, which further improves the activity of the catalytic layer. These findings are consistent with the experimental results.

### 2.6. Electrochemical Stability Test

**Figure 13:**
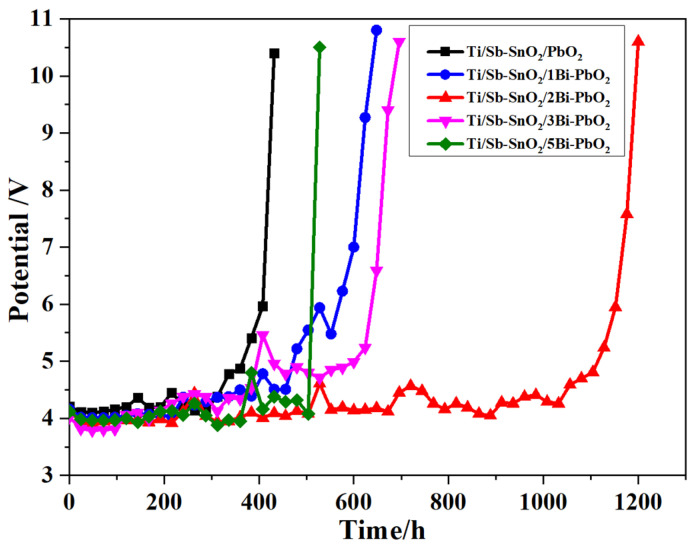
Variation of cell potential with time in the accelerated life test for different electrodes.

**Figure 14:**
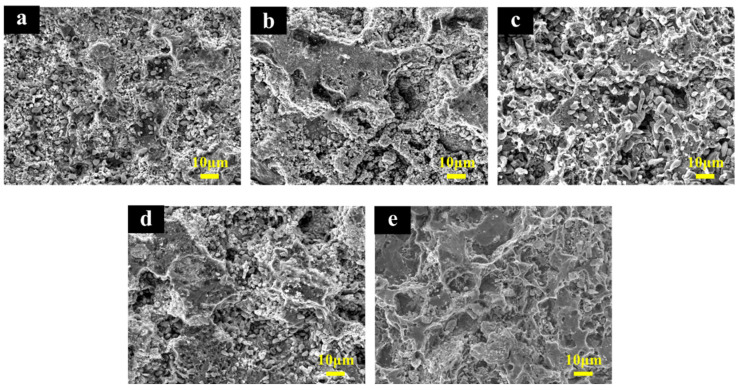
SEM of the deactivated electrodes: (**a**) Ti/Sb-SnO_2_/PbO_2_; (**b**) Ti/Sb-SnO_2_/1Bi-PbO_2_; (**c**) Ti/Sb-SnO_2_/2Bi-PbO_2_; (**d**) Ti/Sb-SnO_2_/3Bi-PbO_2_; (**e**) Ti/Sb-SnO_2_/5Bi-PbO_2_.

**Figure 15:**
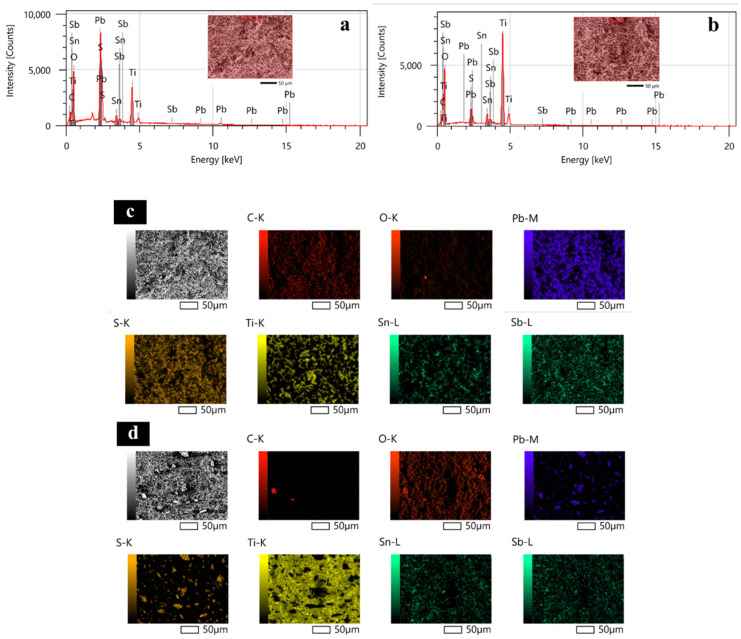
(**a**) the EDS of the deactivated Ti/Sb-SnO_2_/PbO_2_ electrode; (**b**) the EDS of the deactivated Ti/Sb-SnO_2_/2Bi-PbO_2_ electrode; (**c**) EDS-mapping of the deactivated Ti/Sb-SnO_2_/PbO_2_ electrode; and (**d**) EDS-mapping of the deactivated Ti/Sb-SnO_2_/2Bi-PbO_2_ electrode.

The accelerated life tests were carried out to evaluate the electrodes’ stability. Figure 13 displays the time course of cell potential in the accelerated life measurement for the as-prepared electrodes. It is observed that the Ti/Sb-SnO_2_/2Bi-PbO_2_ electrode exhibits the longest lifetime of 1196 h, which is 2.79 times that of the Ti/Sb-SnO_2_/PbO_2_ electrode (428 h). The lifetime of Ti/Sb-SnO_2_/2Bi-PbO_2_ electrodes is longer than those of the other electrodes. This can be attributed to the growth of the PbO_2_ crystal grain, which reduces the gap between the grain boundaries, making the electrode surface denser. The SEM results show that the PbO_2_ particle size becomes small after Bi^3+^ doping. The decrease in the PbO_2_ particle size could reduce the defect density of the electrode surface and make a compact and fine surface layer. The compact electrode surface could not only baffle the penetration of the electrolyte through cracks and pores but also prevent an increase in pressure inside the electrode, caused by the internal oxygen evolution. The lifetime of the Ti/Sb-SnO_2_/3Bi-PbO_2_ electrode is smaller than that of the Ti/Sb-SnO_2_/2Bi-PbO_2_ electrode. This is probably because the nucleation rate is larger than the growth rate of the grain, thereby increasing grain addition. Moreover, the internal stress of the crystal grain is increased, and the surface is cracked. Therefore, the corrosion resistance of the electrode is reduced. The SEM of the as-prepared deactivated electrodes was measured to study the deactivated behavior, as shown in Figure 14. It is observed that the coating of all deactivated electrodes disappeared, and some granular agglomeration was on the electrode. The EDS and EDS-mapping results of the undoped Ti/Sb-SnO_2_/PbO_2_ electrode and the Ti/Sb-SnO_2_/2Bi-PbO_2_ electrode are displayed in Figure 15. It can be seen that Ti, Sn, and Sb elements were detected in both of the deactivated electrodes, indicating that the coating fell off from the electrode surface and that the Sb-SnO_2_ intermediate layer and the Ti substrate were exposed. Moreover, Pb, S, and O elements were also measured. This can be ascribed to the transformation of PbO_2_ to PbSO_4_ during the long-time electrolysis process. From these results, we can speculate that the deactivation of the electrode is due to the detachment of the active layer.

### 2.7. Simulated Zinc Electrowinning Experiment

**Figure 16:**
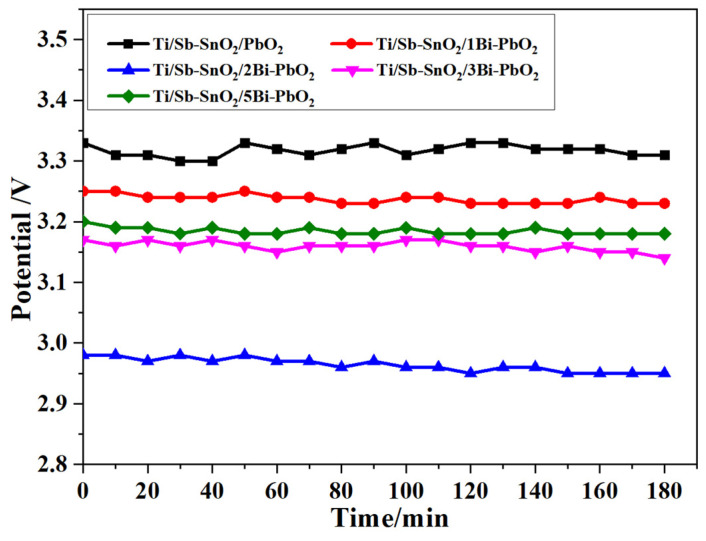
Cell voltage changes at 450 A·m^−2^ during the simulated zinc electrowinning in 3h.

The simulated zinc electrowinning experiment was carried out to study the voltage change of the electrode. Figure 16 shows the cell voltage of the as-prepared electrodes during the 3 h zinc electrowinning process. The cell potential was calculated using the following equation:

U = (E_1_ − E_2_) + IR (8)

where E_1_ − E_2_ is the electrode polarization potential, including the theoretical decomposition voltage and overpotential of ZnSO_4_. R is the total resistance, including the electrode resistance, electrolyte resistance, and contact resistance. The average cell potentials of Ti/Sb-SnO_2_/PbO_2_, Ti/Sb-SnO_2_/1Bi-PbO_2_, Ti/Sb-SnO_2_/2Bi-PbO_2_, Ti/Sb-SnO_2_/3Bi-PbO_2_, and Ti/Sb-SnO_2_/5Bi-PbO_2_ are 3.32 V, 3.24 V, 2.96 V, 3.16 V, and 3.18 V, respectively. It can be seen that the doping of Bi^3+^ reduces the cell voltage of the electrodes. From the Tafel curve, it can be found that the b value decreases with the introduction of Bi^3+^, indicating a small overpotential of the electrode. According to Equation (9), the cell voltage decreases with the decrease in the polarization potential. Moreover, it should be noted that the polarization potential has a positive relationship with the overpotential. Therefore, the decrease in overpotential results in a decrease in voltage drop. Commonly, the small cell potential is favorable for the reduction in energy consumption. The smallest cell voltage was obtained by the Ti/Sb-SnO_2_/2Bi-PbO_2_ electrode, suggesting less energy consumption during zinc electrowinning.

## 3. Experimental Details

### 3.1. Pretreatment of Ti Sheet

The Ti foil (TA1), with a 1.5 mm thickness, was cut into pieces with a dimension of 80 mm × 100 mm before the experiments. The surface of the Ti sheet was subjected to pre-treatment to obtain a gray surface with uniform roughness. The pre-treatment contained degreasing, polishing, and etching for 2 h in boiling oxalic acid. Finally, the specimens were washed with distilled water.

### 3.2. Preparation of Sb-SnO_2_ Interlayer

The sol-gel technique was employed to deposit the Sb-SnO_2_ interlayer to prevent the passivation of the Ti substrate. The citric acid (CA) was dissolved in ethylene glycol (EG) to form citric acid ester, which was then added to SnCl_4_ and SbCl_3_ to obtain sol-gel precursor solutions. The molar ratio of Sn: Sb was 10:1 and the molar ratio of CA: EG was 1:4. The precursor solutions were brushed on the acid-etched Ti sheet and then placed in an oven at 130 °C and annealed at 500 °C. This procedure was repeated several times.

### 3.3. Synthesis of Bi^3+^ Doped Ti/Sb-SnO_2_/PbO_2_ Electrode

Bi^3+^ doped Ti/Sb-SnO_2_/PbO_2_ electrodes were prepared using an anodic electrodeposition approach with a Ti sheet as the cathode. The deposition solutions contained Pb(NO_3_)_2_, Cu(NO_3_)_2_, HNO_3_, some additives, and 0–5 mmol·L^−1^ Bi(NO_3_)_3_. The current density was controlled at 200 A·m^−2^ for 2 h and the bath temperature was 60 °C. Magnetic stirring was employed to enhance the electrolytic diffusion property. The as-prepared electrodes were rinsed thoroughly with deionized water and were denoted as Ti/Sb-SnO_2_/xBi-PbO_2_, where x (x = 1, 2, 3, 5) presents the Bi^3+^ concentration. In addition, the blank sample was also prepared and was marked as Ti/Sb-SnO_2_/PbO_2_.

### 3.4. Characterization and Electrochemical Measurements

The surface morphology and composition of the as-prepared electrodes were characterized using scanning electron microscopy (SEM, JSM-IT200, JEOL, Tokyo, Japan), and the microscope was equipped with an energy-dispersive X-ray spectroscopy (EDS, JEOL, Tokyo, Japan) detector. The crystal pattern of the electrodes was detected by an X’pert PRO MRD diffractometer (XRD, D8 Advance, Bruker, Germany). The chemical states of Pb, O, and Bi in the as-prepared electrodes were determined using X-ray photoelectron spectroscopy (XPS, ESCALAB 250Xi, ThermoFisher, Waltham, MA, USA) on an Ultra DLD Electron Spectrometer (Al Ka radiation; hν = 1486.71 eV). The binding energy of each spectrum was calibrated with C1s (284.8 eV) and was fitted using commercial software (Thermo Avantage 5.9931). Powereach JC2000D was used to measure the contact angle (θ) of the electrode surface. To avoid the effect of the droplet’s gravity on the contact angle, all contact angles were tested 10 s after the droplets were dropped onto the facet. The experiment was conducted at room temperature (25 °C).

An electrochemical workstation (Corrtest CS2350) was adopted to conduct electrochemical research using a traditional three-electrode system. The working electrode was the as-prepared electrode connected to a reference electrode (SCE). The Pt sheet served as the counter electrode. The simulated zinc electrowinning electrolyte was composed of 50 g·L^−1^ Zn^2+^ and 150 g·L^−1^ H_2_SO_4_. The linear sweep voltammetry (LSV) characterization was measured at the scan rate of 10 mV·s^−1^. Cyclic voltammetry (CV) was performed at the scan rate between 10 and 100 mV·s^−1^. The voltammetric charge (q*) was evaluated by integrating cyclic voltammograms. EIS experiments were carried out at 1.9 V in the frequency range of 100 kHz–0.1 Hz with an amplitude of 5 mV. The potential values in this work are all quoted with respect to Ag/AgCl. Accelerated life tests were carried out to research the stability and lifetime of the as-prepared electrodes in 15% H_2_SO_4_ aqueous solutions with a current density of 10,000 A·m^−2^. In this procedure, the experiment is supposed to be finished when the cell voltage is higher than 10 V. Moreover, to study the anodic performance and durability of the composited electrodes, galvanostatic polarization was tested under the simulated zinc electrowinning condition at a current density of 450 A·m^−2^.

### 3.5. Theoretical Calculation

First-principle calculations were conducted on the Vienna Ab initio Simulation Package (VASP (6.X.X.)) [45]. The exchange-correlation influences are described by the Perdew-Burke-Ernzerhof (PBE) functional within the generalized gradient approximation (GGA) method [46]. The core-valence interactions were accounted for using the projected augmented wave (PAW) method [47]. The simulation model was built based on Visualization for electronic and structural analysis (VESTA (3.5.8)) software. The simulation model for Bi-PbO_2_ was built by uniformly doping Bi atoms into PbO_2_ (110) fragments. The energy cutoff for plane wave expansions was set to 480 eV, and the 3 × 3 × 1 Monkhorst-Pack grid k-points were selected to sample the Brillouin zone integration. The vacuum space was adopted 15 Å above the surfaces to avoid periodic interactions. The structural optimization was completed for energy and force convergence set at 1.0 × 10^−4^ eV and 0.02 eV Å^−1^, respectively.

The Gibbs free energy change (ΔG) of each step was calculated using Formula (1)

∆G = ∆E + ∆ZPE − T∆S (9)

where ΔE is the electronic energy difference directly obtained from DFT calculations; ΔZPE is the zero point energy difference; T is the room temperature (298.15 K); and ΔS is the entropy change.

## 4. Conclusions

In this study, we studied the role of Bi(NO_3_)_3_ doping in enhancing the electrochemical performance of Ti/Sb-SnO_2_/PbO_2_ electrodes in zinc electrowinning. The XPS measurement confirms that Bi^3+^ is successfully doped into the electrode. The addition of Bi(NO_3_)_3_ could refine the surface grain, change crystal structure, improve the hydrophilicity, and enhance the electrocatalytic activity of the Ti/Sb-SnO_2_/PbO_2_ electrode. We found that the Bi(NO_3_)_3_ content has a significant influence on the coating morphology, the crystal orientation, the surface hydrophilicity, the oxygen evolution reaction kinetics, the charge-transfer efficiency, the stability, and the reactivity. The hydrophilicity, the OEP, and the R_ct_ exhibit a declining trend when there is an increase in the Bi(NO_3_)_3_ concentration. The optimum electrocatalytic activity of the Ti/Sb-SnO_2_/Bi-PbO_2_ electrode is achieved when the concentration of Bi(NO_3_)_3_ is 2 mmol·L^−1^. The Ti/Sb-SnO_2_/2Bi-PbO_2_ electrode illustrates the largest q_T_* of 21.20 C·cm^−2^ and the lowest overpotential of 0.814V (500 A·m^−2^), suggesting that it has the more active site and the higher oxygen evolution activity. Our DFT calculations demonstrate that the introduction of Bi^3+^ leads to a decrease in the OER reaction barrier and an increase in the DOS of the electrode, which further enhances the catalytic activity and conductivity of the electrode. This theoretical calculation is consistent with the experimental results. Furthermore, the accelerated life measurement indicates that the introduction of Bi^3+^ can enhance the stability of the electrode, and that the Ti/Sb-SnO_2_/2Bi-PbO_2_ electrode possesses the highest accelerated lifetime of 1196 h, which is 2.79 times that of the undoped electrode. The reason for the failure of the electrode is that the active coating gradually detaches from the electrode surface as the electrolysis time increases, until the active layer disappears completely. Furthermore, compared with the undoped electrode, the cell voltage of the Ti/Sb-SnO_2_/2Bi-PbO_2_ electrode is reduced, indicating less energy consumption. In summary, the proper doping of Bi^3+^ is beneficial for improving the electrocatalytic activity of the Ti/Sb-SnO_2_/PbO_2_ electrode. These attractive results show that the potential future applications of this electrode material should be strongly anticipated in zinc electrowinning.

## Figures and Tables

**Table 1:** The binding energy of O 1s and its proportions on each electrode.

Electrode	O_L_/eV	O_L_/%	O_d-ad_/eV	O_d-ad_/%	O_s-ad_/eV	O_s-ad_/%
Ti/Sb-SnO_2_/PbO_2_	529.16	36.4	530.85	35.8	532.65	27.8
Ti/Sb-SnO_2_/1Bi-PbO_2_	529.11	26.6	531.48	47.3	532.95	26.1
Ti/Sb-SnO_2_/2Bi-PbO_2_	529.12	28.5	530.74	51.5	533.07	20.0
Ti/Sb-SnO_2_/3Bi-PbO_2_	529.18	32.8	530.68	50.3	533.12	16.8
Ti/Sb-SnO_2_/5Bi-PbO_2_	529.22	25.4	531.04	41.6	532.97	33.0

**Table 2:** The binding energy of Pb 4f on each electrode.

Electrode	Pb^4+^4f_7/2_/eV	Pb^4+^4f_5/2_/eV	Pb^2+^4f_7/2_/eV	Pb^2+^4f_5/2_/eV
Ti/Sb-SnO_2_/PbO_2_	137.20	142.01	137.96	142.82
Ti/Sb-SnO_2_/1Bi-PbO_2_	137.07	141.92	137.80	142.67
Ti/Sb-SnO_2_/2Bi-PbO_2_	137.06	141.96	137.79	142.74
Ti/Sb-SnO_2_/3Bi-PbO_2_	137.15	142.04	137.88	142.83
Ti/Sb-SnO_2_/5Bi-PbO_2_	137.29	142.06	138.17	142.92

**Table 3:** The equivalent circuit parameters of the as-prepared electrodes.

Materials	R_s_/ohm	R_ct_/ohm	CPE/S·sec^^n^·cm^−2^	n
Ti/Sb-SnO_2_/PbO_2_	1.521	20.80	0.0306	0.921
Ti/Sb-SnO_2_/1Bi-PbO_2_	0.797	16.07	0.0369	0.914
Ti/Sb-SnO_2_/2Bi-PbO_2_	0.952	7.30	0.0599	0.905
Ti/Sb-SnO_2_/3Bi-PbO_2_	0.854	11.29	0.0388	0.908
Ti/Sb-SnO_2_/5Bi-PbO_2_	0.823	14.37	0.0350	0.920

**Table 4:** Overpotential and kinetic parameters for oxygen evolution on different samples.

Materials	a/V	b/V	η	
			500 A·m^−2^	1000 A·m^−2^
Ti/Sb-SnO_2_/PbO_2_	1.141	0.187	0.898	0.954
Ti/Sb-SnO_2_/1Bi-PbO_2_	1.091	0.171	0.869	0.920
Ti/Sb-SnO_2_/2Bi-PbO_2_	1.023	0.161	0.814	0.862
Ti/Sb-SnO_2_/3Bi-PbO_2_	1.096	0.164	0.882	0.931
Ti/Sb-SnO_2_/5Bi-PbO_2_	1.114	0.171	0.892	0.944

**Table 5:** The detailed voltammetric charge (q*) of different samples.

Materials	q_T_*/C·cm^−2^	q*_O_/C·cm^−2^	q*_i_/C·cm^−2^	q*_i_/q_T_*
Ti/Sb-SnO_2_/PbO_2_	4.44	0.68	3.76	84.7%
Ti/Sb-SnO_2_/1Bi-PbO_2_	7.25	1.01	6.24	86.1%
Ti/Sb-SnO_2_/2Bi-PbO_2_	21.20	1.09	20.11	94.9%
Ti/Sb-SnO_2_/3Bi-PbO_2_	11.49	1.18	10.31	89.8%
Ti/Sb-SnO_2_/5Bi-PbO_2_	11.01	1.11	9.90	89.9%

## Data Availability

The data is unavailable due to privacy.
